# Severe pneumonia caused by *Legionella pneumophila* associated with *Tropheryma whipplei*: A case report

**DOI:** 10.1097/MD.0000000000043121

**Published:** 2025-07-11

**Authors:** Dian Jin, Fang Li, Jing Fang, Jun Wang, Song-Tao Li

**Affiliations:** a Department of Pharmacy, Chengdu Sixth People’s Hospital, Chengdu, China; b Department of Pulmonology, Chengdu Sixth People’s Hospital, Chengdu, China.

**Keywords:** delirium, *Legionella pneumophila*, Liver Function Abnormalities, pharmacological monitoring, *Tropheryma whipplei*

## Abstract

**Rationale:**

*Legionella pneumophila* (*LP*) and *Tropheryma whipplei* (*TW*) have been identified as pathogens that can coexist in sewage environments, which can both cause pneumophila and can impact various organs, leading to extrapulmonary manifestations. It is rare to find literature on pneumonia caused by atypical pathogens, such as the combination of *LP* and *TW*. There are no reports on specific drug treatments for such combined infections.

**Patient concerns:**

A 55-year-old male with no underlying diseases developed community-acquired pneumonia. His condition worsened after empirical treatment with piperacillin/tazobactam. He subsequently experienced adverse drug reactions after using moxifloxacin.

**Diagnoses:**

Pulmonary infection, pulmonary bullae.

**Interventions:**

After admission, the patient was treated with piperacillin/tazobactam and moxifloxacin for anti-infection. He also underwent a bronchoscopy of the lungs, and the alveolar lavage fluid was tested using targeted next-generation sequencing. The results identified pathogens such as *LP* and *TW*. The anti-infection regimen was then adjusted to a combination of ceftriaxone and levofloxacin. Symptomatic treatments were also provided, including expectorant therapy, liver protection, and nutritional support.

**Outcomes:**

The patient was discharged after showing signs of improvement and was subsequently monitored at an outpatient clinic.

**Lessons:**

When empirical antibiotic therapy for pneumonia is ineffective, infections caused by atypical pathogens should be considered. To prevent progression to severe pneumonia and even patient death, the precise use of antibiotics is beneficial for improving patient outcomes. However, the diagnosis of atypical pathogens is challenging, and the selection of drugs also needs to be considered from multiple aspects.

## 1. Introduction

Legionnaires’ disease, also known as *Legionella pneumophila pneumonia* (*LPP*), is a respiratory infection caused by the bacterium *LP*.^[1]^ *Legionella* spp. predominantly inhabits natural freshwater and artificial water settings, with its primary mode of transmission being through the air. Human infection and disease manifestation occur following the inhalation of aerosols carrying *Legionella* bacteria.^[2]^ This disease can manifest in various organs in the body,^[3]^ presenting clinical symptoms such as chills, fever, dry cough, and respiratory distress as pulmonary symptoms, and diarrhea, delirium, and hypokalemia as extrapulmonary symptoms.^[4,5]^ *LPP* is often misdiagnosed or overlooked, progressing rapidly compared to other atypical pathogen pneumonias, leading to severe pneumonia, respiratory failure, shock, acute renal failure, and multiple organ dysfunction.^[6]^ The mortality rate ranges from 4% to 18%.^[7]^ Whipple disease (WD) is a rare condition with an incidence rate of about 1/1,000,000, and the average age at diagnosis is 55 years, with 85% of patients being male.^[8]^ This disease often affects multiple organs and systems throughout the body, with the classic triad of symptoms being arthritis, digestive system symptoms, and weight loss.^[9]^ Additionally it may present with neurological symptoms, cardiovascular issues^[10]^ as well as chronic localized infections in other tissues^[11]^ and acute infections.^[12]^ WD is not commonly seen in clinical practice, and the lack of specificity in laboratory and imaging tests makes its diagnosis of WD relatively difficult. In this case, the pathogen was rapidly and accurately identified as *LP* and *TW* through targeted next-generation sequencing (tNGS) technology. The purpose of this case report is to provide insights into diagnostic methods and treatment options to guide clinical decision-making.

## 2. Case description

**Figure 1.**
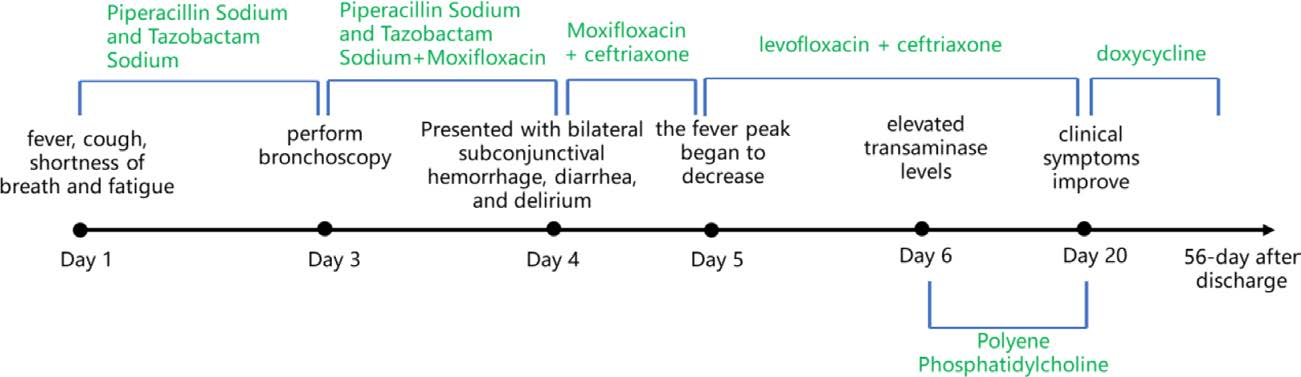
Timeline of the hospitalization and treatment course.

A 55-year-old male with no significant health conditions and a history of exposure to sewage environments was admitted to the hospital due to a 3-day history of cough and fever, accompanied by dyspnea and generalized body aches. Based on the results of a chest computed tomography scan, which revealed pulmonary inflammatory lesions, along with elevated serum inflammatory markers, the initial diagnosis upon admission was a pulmonary infection. Empirical treatment with piperacillin sodium and tazobactam sodium was initiated. However, after 2 days of treatment, there was no significant improvement in his condition, and he remained febrile, prompting the addition of moxifloxacin 0.4 g once daily to cover atypical pathogens. The following day, the patient developed symptoms including bilateral conjunctival patchy bleeding, diarrhea, and delirium. Sputum smears, cultures, and bronchial lavage fluid smears and cultures were all negative, while the tNGS test on the bronchoalveolar lavage fluid (BALF) identified *LP*, *TW*, and *Fusobacterium nucleatum* (*FN*). Based on the tNGS sequencing results, the pathogens considered to be *LP* and *TW*, which leading to the diagnoses of *LPP* and *TW* pneumonia. The patient’s clinical symptoms further supported these diagnoses. The antibiotic treatment plan was adjusted to include levofloxacin 0.5 g once daily and ceftriaxone 3 g once daily, resulting in a marked improvement in the patient’s condition. He was eventually discharged with instructions to continue oral doxycycline 0.1 g twice daily. The patient had 4 follow-up appointments after discharge, demonstrating continued improvement in their condition and no significant adverse drug reactions. The length of the hospital stay and the treatment process are illustrated in Figure 1, and a detailed case progress report is provided below.

### 2.1. Patient information

The patient is a 55-year-old male with no known underlying health conditions. He presents with a dry cough, accompanied by chills and fever, with a maximum body temperature of 39.2°C. Additionally, he reports experiencing chest tightness, dyspnea, and generalized body aches. He self-administered the traditional Chinese medicine for fever over the course of 3 days without any improvement in symptoms. The patient has a history of exposure to sewage at a shipyard and denies any significant family medical history.

### 2.2. Physical examination

Temperature: 39.2°C, pulse: 141 beats/min, Respiration: 20 breaths/min, blood pressure: 103/74 mm Hg. The patient is conscious and responsive, with auscultation revealing moist rales in the left lower lung.

### 2.3. Laboratory and imaging examinations

The laboratory tests revealed the following results: white blood cell count of 17.77 × 10^9^/L, neutrophil percentage (NEUT%) of 88.5%, procalcitonin level of 2.12 ng/mL, high-sensitivity C-reactive protein level of 290.39 mg/L, creatinine (Cr) level of 86.5 μmol/L, alanine aminotransferase level of 17 U/L, and aspartate aminotransferase level of 20 U/L(Table 1).

**Table 1:** Changes in inflammatory indexes and liver and kidney functions of patients.

Time	Tmax (°C)	WBC × 10^9^/L	NEUT% (%)	hsCRP (mg/L)	PCT (ng/mL)	ALT (U/L)	AST (U/L)	Cr (µmol/L)
7/13/2024	39.2	17.77	88.5	290.39	2.12	17	20	86.5
7/16/2024	39.9	6.18	91.7	276.78	7.492	25	36	74.1
7/17/2024	39	6.08	89.3	272.72	5.385	33	48	66.5
7/18/2024	37.9	3.44	77.2	247.84	3.636	70	98	58.9
7/20/2024	36.7	4.5	76.2	111.62	1	83	81	53.2
7/24/2024	36.4	8.37	73.4	63.28	0.191	67	47	55.1
7/31/2024	36.6	5.63	51.4	17.42	–	36	23	58.9
8/8/2024	36.3	–	–	–	–	19	22	59.9
8/15/2024	36.6	6.68	54.3	–	–	–	–	–
8/22/2024	36.5	8.98	53.9	24.58	–	24	25	60.8
9/26/2024	36.4	7.09	51	2.71	–	–	–	42.8

ALT = alanine transaminase, AST = aspartate transaminase, Cr = creatinine, hsCRP = hypersensitive C-reactive protein, NEUT% = neutrophil percentage, PCT = procalcitonin, WBC = white blood cells.

**Figure 2.**
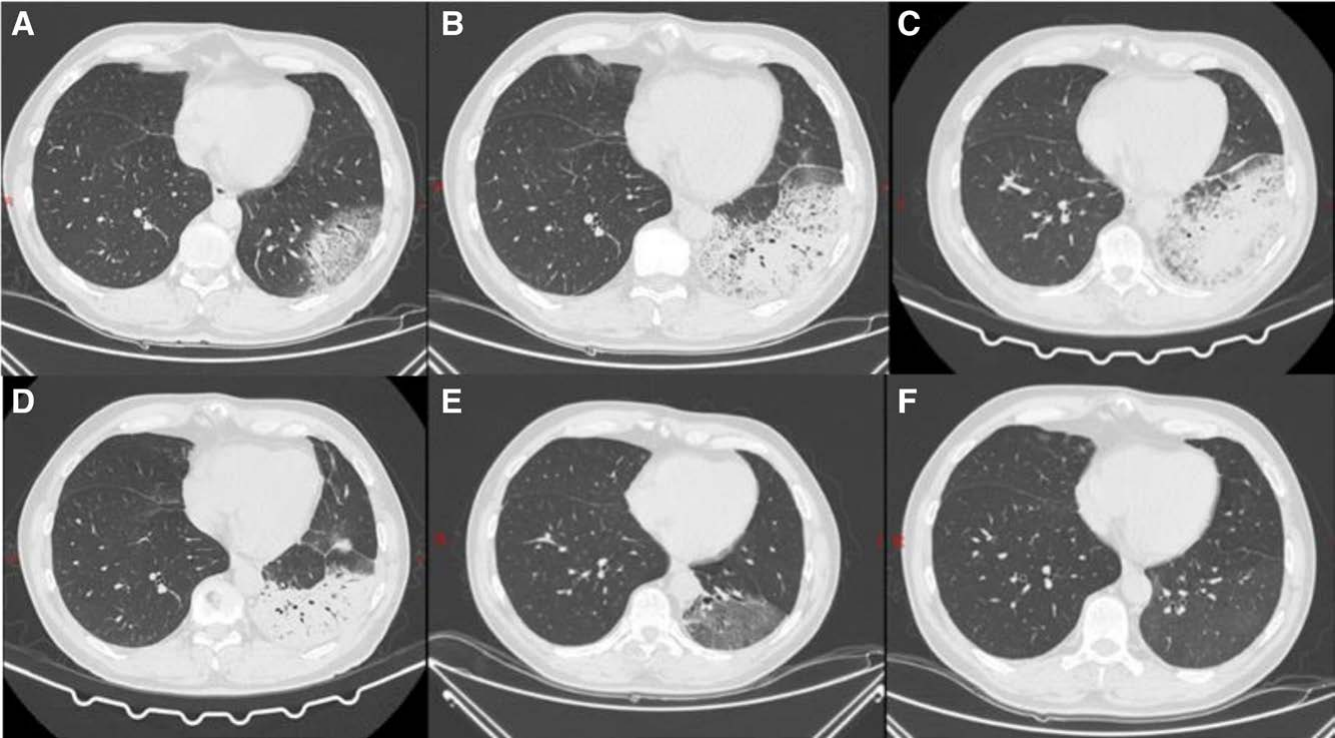
Chest computed tomography image. (A) At admission (7/13/2024), a large area of ground-glass density-increased shadow in the lower lobe of the left lung is grid-like, mainly characterized by inflammation and interstitial inflammation. (B) On day 4 (7/16/2024), there is a large area of inflammatory foci in the lower lobe of the left lung, with partial consolidation. The inflammation has significantly increased in extent compared to the previous imaging. A small amount of inflammatory foci is also noted in the right lung. (C) On day 11 (7/23/2024), there were large inflammatory foci in the lower lobe of the left lung, some of which were consolidated. Compared with before, the lesion density decreased and the range slightly narrowed. A few blurred flocculent shadows were newly added in the interlobar pleural cavity adjacent to the upper lobe of the left lung, which were newly added inflammatory lesions. A few inflammatory foci in the right lung have a higher density than before, and the subpleural interstitium in the lower lobe of the right lung is thickened. (D) On day 19 (7/31/2024), there are large inflammatory foci in the lower lobe of the left lung, with partial consolidation. Compared with before, the lesion density has increased and the range has slightly narrowed. A few indistinct flocculent shadows in the interlobar pleural cavity adjacent to the upper lobe of the left lung are inflammatory lesions, with reduced absorption compared to before. A few inflammatory foci in the right lung, basically the same as before, and thickening of the subpleural interstitium in the lower lobe of the right lung. (E) At follow-up 31 days after discharge (9/1/2024), there were large inflammatory foci in the lower lobe of the original left lung, with partial consolidation. Compared with before, the lesion density became lighter and the range significantly narrowed. A few new inflammatory foci were added in the upper lobe of the left lung. A few inflammatory foci in the right lung were absorbed more than before. (F) At follow-up 56 days after discharge (9/26/2024), the inflammatory foci in the upper and lower lobes of the original left lung have partially consolidated. Compared with before, the absorption of the lesion has significantly decreased, and currently there is a small residual ground-glass density shadow.

Chest CT reveals a large area of increased ground-glass opacity in the left lower lobe of the lung, with a reticular pattern, predominantly indicating inflammation and interstitial inflammation (Fig. 2A).

### 2.4. Microbiological examinations

On admission, sputum samples from the patient were cultured for bacteria and fungi, with negative results, and acid-fast staining for tuberculosis was also negative; serum antibodies for respiratory pathogens were tested, with negative outcomes as well. On the third day of hospitalization, the patient underwent bronchoscopy, and the lungs were lavaged with 50 mL of 0.9% sodium chloride injection solution, from which BALF samples were obtained for tNGS and microbial culture. The microbial culture of the BALF remained negative. tNGS detection was carried out with the KM MiniseqDx-CN system (20203220340). The BALF tNGS results identified *LP* with 38,186 sequences, *TW* with 9463 sequences, and *FN* with 162 sequences (Table 2).

**Table 2:** Pathogenic microorganism detection results.

Microorganism type	Genus	Microorganism name	Normalized sequence count	Estimated microbial concentration (copies/mL)	Pathogenicity classification
1. Special pathogen list (mycobacteria, mycoplasmas, chlamydiae, etc)					
Not detected					
2. Bacterial list					
Gram negative bacteria	Legionella	*Legionella pneumophila*	38186	>1.0 × 10^6^	A
Gram positive bacteria	Whipplei	*Tropheryma whipplei*	9463	>1.0 × 10^6^	B
Gram negative bacteria	Fusobacterium	*Fusobacterium nucleatum*	162	<1.0 × 10^3^	C
3. Fungal list					
Not detected					
4. Viral list					
Not detected					

### 2.5. Treatment and pharmaceutical care

On the first day (7/13/2024) following the patient’s admission, a chest CT scan (Fig. 2A) revealed inflammatory changes in the lungs, elevated inflammatory markers, and blood gas analysis indicated low partial pressure of oxygen. The patient was empirically treated with piperacillin sodium and tazobactam sodium (4.5 g), which was added to 100 mL of 0.9% sodium chloride injection and administered intravenously every 8 hours. After 2 days of treatment, the patient continued to experience fever, with an increased peak temperature, reaching a maximum of 39.4°C. The clinical pharmacist assessed that the anti-infective response was inadequate, noting that atypical pathogens were not adequately covered. Consequently, moxifloxacin (0.4 g qd) was added to the treatment regimen, and the physician accepted this recommendation.

On the third day (7/15/2024), the patient continues to present with persistent fever, with no reduction in peak body temperature. As the sputum smear and culture results remain pending and empirical anti-infective therapy has shown suboptimal efficacy, we have decided to perform bronchoscopy to exclude other diseases with similar symptoms. Concurrently, bronchoalveolar lavage will be conducted, and the lavage fluid will undergo tNGS testing. Compared to conventional sputum cultures or bronchial lavage fluid cultures, tNGS offers higher sensitivity, faster turnaround time, and unbiased detection of a broad spectrum of pathogenic microorganisms. This approach enables precise antimicrobial therapy based on the diagnostic findings.

On the fourth day (7/16/2024), the patient’s inflammatory markers decreased; however, clinical symptoms worsened, manifesting as an increased peak fever, worsening cough and sputum production, new bilateral conjunctival hemorrhages, diarrhea, and delirium. A repeat chest CT scan (Fig. 2B) revealed a large inflammatory focus in the left lower lobe of the lung, which had significantly increased compared to the previous scan. BALF tNGS indicated the following sequence counts: *Legionella pneumophila* (*LP*) 38,186, *Tropheryma whipplei* (*TW*) 9463, and *FN* 162. The antibiotic treatment plan was adjusted to include levofloxacin 0.5 g once daily, combined with ceftriaxone 3 g once daily.

On the sixth day (7/18/2024), the patient’s inflammatory markers decreased, and clinical symptoms improved; however, transaminases increased. Therefore, polyene phosphatidylcholine 456 mg was added 3 times a day for liver protection.

After 15 days of adjusted antibiotic treatment, the patient exhibited no significant cough or sputum production, no further fever, normal inflammatory markers, and normal liver and kidney function. A chest CT scan (Fig. 2C) revealed a reduced lesion area, and the patient was discharged, continuing to take doxycycline 0.1 g twice a day as part of the treatment plan.

Follow-up visits on the 31st and 56th days after discharge revealed normal liver and kidney function. The most recent chest CT scan (Fig. 2D) indicated a significant reduction in the lesion, with only a few remaining ground-glass opacities; consequently, the medication was discontinued.

## 3. Discussion

### 3.1. Identification of pathogenic bacteria

The patient is a middle-aged male with no underlying medical conditions and an acute onset. Chest CT upon admission showed no halo sign or cavitation. Given the lesion is localized to the left lower lobe rather than involving both lungs, fungal infections, tuberculosis, and viral etiologies are considered less likely, with bacterial infection being the predominant diagnostic consideration. After 2 days of empirical antibacterial treatment with no significant improvement in clinical symptoms, we considered the possibility of infection caused by atypical pathogens. For patients suspected of atypical pathogen infection, obtaining a detailed medical history is fundamental. Clinicians should focus on inquiring about animal exposure history, occupational characteristics, and travel/residence history to preliminarily differentiate the category of atypical pathogens. The patient had a history of exposure to sewage from a shipyard, so we suspected that he might have been infected with *Legionella*. The standard method for diagnosing *Legionella* bacteria infection involves the isolation and culture of the bacteria from specimens, primarily obtained from the patient’s lower respiratory tract secretions, such as sputum and bronchoalveolar lavage fluid. However, the positive detection rate is relatively low.^[13]^ Highly suspicious of *Legionella* bacteria infection, urine antigen testing^[14]^ or PCR^[15]^ can be carried out, this can shorten the time to diagnosis. The patient underwent multiple sputum cultures during their hospital stay, all of which returned negative results. Additionally, the BALF culture did not isolate *LP*. Whipple disease is easily missed at the initial stage. PAS staining is regarded as the standard method for diagnosing WD.^[16]^ However, the patient did not exhibit prominent gastrointestinal symptoms, hence this testing method was not employed. Although pulmonary involvement by *TW* is rare, the elevated sequence count of *TW* detected via tNGS in this case suggests its potential role as one of the pathogens contributing to this severe pneumonia episode. tNGS combines targeted amplification with NGS technology. This approach enables the detection of both common and rare pathogens, independent of culture methods. Additionally, it minimizes interference from the host and background, while simultaneously enhancing the detection rate of low-abundance pathogens.^[17,18]^ The detection rate for dual mixed infections and triple or more mixed infections is higher than that of traditional methods.^[19]^ This case report utilized tNGS to identify the causative pathogens, *LP* and *TW*, thereby assisting clinical physicians in selecting appropriate antimicrobial agents and preventing the worsening of the condition.

### 3.2. Anti-infection treatment plans

In accordance with established guidelines,^[20]^ the recommended treatment for *LPP* includes quinolone antibiotics like levofloxacin, moxifloxacin, or macrolide antibiotics such as azithromycin. Studies have shown that quinolones exhibit superior activity against *LP* compared to macrolides.^[21]^ Consequently, the clinical pharmacist advised prioritizing the use of quinolone antibiotics. *TW* is a gram positive rod-shaped bacterium classified under the family Coriobacteriaceae^[22]^ and is known to cause WD. Currently, there are no established guidelines or expert consensus on the treatment of WD. However, literature suggests treatment options such as doxycycline in combination with hydroxychloroquine^[23]^ or the sequential administration of ceftriaxone or meropenem with co-trimoxazole.^[24]^ To avoid the adverse effects of bacterial resistance caused by the use of broad-spectrum antibiotics, clinical pharmacists recommend the use of the narrow-spectrum antibiotic ceftriaxone. The patient received treatment with levofloxacin 0.5 g once daily in combination with ceftriaxone 3 g once daily for anti-infection purposes. The patient’s inflammatory markers significantly decreased and clinical symptoms improved with the anti-infective treatment of levofloxacin combined with ceftriaxone, indicating that the anti-infective regimen was effective.

The standard treatment duration for *LPP* typically spans from 14 to 21 days, contingent upon the patient’s immune function status.^[7]^ Conversely, the treatment duration for WD varies significantly, ranging from 4 days to 6.5 months^[25]^ Doxycycline is effective in treating infections caused by *LPP*^[26]^ and is also active against WD.^[8]^ Consequently, post-discharge, the patient has been advised to continue taking oral doxycycline at a dosage of 0.1 g every 12 hours. On the 31st and 56th days following discharge, follow-up chest CT scans (Fig. 2F) revealed a consistent reduction in the lesions. Consequently, on the 56th day after discharge, taking into account the patient’s symptoms, inflammatory markers, and chest CT findings, the medication was discontinued.

### 3.3. Analysis of the causes of the patient’s mental abnormalities

*LP* has the potential to induce neurological symptoms, such as memory impairment and mental disorders, and can even result in cerebellar dysfunction. The pathogenesis of these symptoms involves immune-mediated mechanisms and neurotoxins.^[27]^ *TW* is another pathogen that can impact the nervous system, commonly leading to cognitive alterations, mental symptoms, and hypothalamic involvement.^[8,28]^ Hence, it is plausible that the patient’s mental abnormalities are among the extrapulmonary symptoms arising from the co-infection of *LP* and *TW*. Upon reviewing relevant materials, neurological adverse reactions are one of the common adverse effects of quinolone antibiotics, but the incidence is relatively rare for levofloxacin compared to moxifloxacin.^[29]^ Due to the fluorine atom in the structure of moxifloxacin, its lipophilicity is increased, making it more likely to penetrate the blood–brain barrier and stimulate the central nervous system.^[30]^ In addition, meropenem may also cause neurological adverse reactions.^[31]^ Therefore, to avoid further exacerbating the patient’s delirium symptoms, we should choose drugs with fewer neurological adverse reactions and continuously monitor the patient’s neurological symptoms during the treatment period.

### 3.4. Analysis of the causes of the patient’s liver function abnormalities

On the 6th day after the patient was admitted, an increase in serum transaminases was observed. Upon reviewing the medical records, it was discovered that the liver enzyme abnormalities appeared 2 days after the administration of ceftriaxone. The Naranjo assessment scale scored 2 points, indicating a potential adverse reaction to ceftriaxone. *LP* can lead to liver function abnormalities, and the patient had already shown symptoms of diarrhea before receiving ceftriaxone. Additionally, since admission, the serum transaminases have been progressively increasing. With no history of hepatitis in the past, it is plausible that the liver function abnormalities are linked to *Legionella* bacteria.^[32]^ The clinical pharmacist believes that the benefits of continuing to use ceftriaxone for this patient are significantly greater than the risks associated with adverse reactions, so there is no need to discontinue the medication. After 14 days of symptomatic liver protection treatment, the serum transaminases returned to normal values.

### 3.5. Analysis of the causes of the patient’s eye redness

*TW* may cause of ocular lesions, manifesting as nonspecific indicators of intraocular inflammation and may precede neurological manifestations.^[33]^ The patient demonstrated subconjunctival congestion on July 15, 2024, followed by symptoms of delirious speech on July 16, 2024, in line with documented cases. Following a 7-day course of ceftriaxone treatment targeting *TW*, the patient’s symptoms of conjunctival congestion showed improvement.

## 4. Conclusion

The patient was infected with 2 atypical pathogens: *LP* and *TW*. If not diagnosed and treated promptly, these infections can progress to severe pneumonia or even become life-threatening within a short period. Traditional culture methods present significant challenges in diagnosing these specific pathogens, specific antigen detection or PCR can simplify the diagnosis, but when clinical manifestations, laboratory indicators, and imaging lack specificity, this diagnostic method is prone to yielding negative results. It is important to choose the appropriate pathogen detection method in clinical practice. tNGS can rapidly and accurately identify the causative agents, especially in cases of mixed infections with multiple pathogens. Therefore, when empirical use of anti-infective drugs proves ineffective, it is essential to consider infections caused by atypical pathogens and to quickly identify the pathogens in order to initiate targeted medication. In terms of drug selection, we should prioritize narrow-spectrum antibiotics once the pathogen is identified. The adverse reactions and therapeutic effects of quinolone drugs presented contradictory challenges in this treatment plan, and the concurrent use of cephalosporin antibiotics also led to drug-related adverse reactions. Therefore, in selecting a medication, we chose levofloxacin, which is effective against *LP* and has minimal effects on the nervous system. We combined it with ceftriaxone, which has a relatively narrow antibacterial spectrum against *TW*. In light of the adverse reaction of elevated transaminases, we conducted a comprehensive assessment of the therapeutic benefits and risks, ultimately deciding to continue ceftriaxone while adding polyene phosphatidylcholine for liver protection. As a result, both the adverse reactions and clinical symptoms improved. When treating pulmonary infections caused by multiple pathogens, it is essential to consider various factors, including patient symptoms, inflammatory markers, and lung CT scans. Medication can be discontinued once all these factors return to normal levels.

## Author contributions

**Conceptualization:** Song-Tao Li.

**Methodology:** Jun Wang.

**Supervision:** Jing Fang.

**Writing – original draft:** Dian Jin.

**Writing – review & editing:** Dian Jin, Fang Li.
